# Expression of Anti-Müllerian Hormone and Its Type 2 Receptor in the Ovary of Pregnant and Cyclic Domestic Cats

**DOI:** 10.3390/ani12070877

**Published:** 2022-03-30

**Authors:** Nilgün Gültiken, Murat Yarim, Selim Aslan, Hande Gürler, Gul Fatma Yarim, Müge Tuncay, Sinem İnal, Sabine Schäfer-Somi

**Affiliations:** 1 Department of Obstetrics and Gynecology, University of Ondokuz Mayis, Samsun 55200, Turkey; vetnilgun@gmail.com (N.G.); handeulusoy@gmail.com (H.G.); ufem1234@gmail.com (M.T.); 2 Department of Pathology, University of Ondokuz Mayis, Samsun 55200, Turkey; myarim@omu.edu.tr (M.Y.); sinem.inal@omu.edu.tr (S.İ.); 3 Department of Obstetrics and Gynecology, Faculty of Veterinary Medicine, Near East University, Nicosia 99138, Cyprus; selim.aslan@neu.edu.tr; 4 Department of Biochemistry, Faculty of Veterinary Medicine, University of Ondokuz Mayis, Samsun 55200, Turkey; gulyarim@omu.edu.tr; 5 Platform for Artificial Insemination and Embryo Transfer, University of Veterinary Medicine, 1210 Vienna, Austria

**Keywords:** cats, pregnancy, estrus, ovary, AMH, AMH receptor

## Abstract

**Simple Summary:**

In female cats, AMH is secreted by ovarian follicles and exerts its effects via its specific receptor AMHRII. Its main function is regulation of cell proliferation and inhibition of excess recruitment of primordial follicles. Since the serum concentration is typically elevated in intact cats, multiple studies have investigated the usefulness of AMH measurement to assess intactness or for diagnosis of ovarian remnant syndrome. However, no study is available concerning the expression and regulation of AMH and AMHRII in feline ovaries. In this study, the site and degree of expression was investigated by using western blot and immunohistochemistry and related to follicle and corpora lutea number, as well as serum AMH concentration in estrus and interestrus cats, during different pregnancy stages.

**Abstract:**

To evaluate the expression of AMH and its receptor AMHRII, ovaries of 33 p cats were investigated by western blot and immunohistochemistry. After ovariohysterectomy, the cats were grouped according to pregnancy stages and ovarian/placental endocrine activity: group I (*n* = 3, 24–29 days), II (*n* = 8, 32–40 days), III (*n* = 4, 41–46 days), IV (*n* = 6, 53–61 days) and according to cycle stages: V (*n* = 6, interestrus) and VI (*n* = 6, estrus). Serum progesterone- and AMH-concentration was measured. Follicle numbers did not differ between groups. The number of corpora lutea was higher in pregnant cats than in the non-pregnant cats. Serum AMH concentration was at maximum between day 30 and 50 of gestation, and was higher than in non-pregnant cats, then decreased towards term (*p* < 0.05). In the ovaries, AMH immunopositivity was observed in granulosa cells of secondary and antral follicles, and in interstitial cells of corpora lutea; highest percentage of immunopositive areas was detected in group III (*p* < 0.05). A positive correlation between the number of corpora lutea and the positive AMH signals in ovarian tissue was determined (r^2^ = 0.832, *p* < 0.05); however, only during mid-gestation (group II). Expression of AMHRII was in close co-localization with AMH and strong in the interstitial cells surrounding follicles undergoing atresia. AMHRII expression did not differ between pregnant groups but was higher compared to estrus cats (*p* ˂ 0.05). We conclude that AMH and AMHRII expression in the feline ovary is comparable to other species. The high serum AMH concentration and ovarian AMHRII expression between day 30 and 50 of gestation are probably related to ovarian activity and follicular atresia.

## 1. Introduction

The glycoprotein Anti-Müllerian Hormone (AMH) or Müllerian Inhibiting Substance is a member of the transforming growth factor-ß superfamily [1]. Members of this family show a high degree of homology among different species. The functions during male and female embryogenesis are well known [2]; AMH participates in cell growth, adhesion, mesenchymal-epithelial interactions, cell differentiation, and apoptosis.

In human medicine, AMH is of great interest as a marker for functional ovarian reserve, comprising the growing follicles capable of ovulation [3]. In men, variable serum concentrations were found in individuals. In general, levels are lower in older men [4] and variable in men suffering from fertility related disorders; measurement of AMH may support the diagnosis of persistent Müllerian duct syndrome [5]. Similarly, in stallions from the age of two years on, measurement of AMH may help to identify disturbances of testicular development [6].

In domestic animals, AMH is currently under investigation as a marker for fertility. In cattle, the AMH serum concentrations are positively related to pregnancy rates and the response to superovulation [7]. In wild species, AMH is gaining interest as a marker for reproductive senescence; a relation between ovarian reserve and serum AMH concentration was found in monkeys [8].

The ovarian regulation of AMH and its specific receptor AMHRII in female mammals is under intense investigation. AMH is produced in the ovaries, by preantral and early antral follicles, and its secretion is stimulated by FSH. In a previous study, gene expression of both AMH and AMHRII was increased after stimulation of cultured bovine granulosa cells with low doses of FSH [9]. However, in cats, no difference was found between serum AMH concentration, before and after intramuscular injection of 0.4 µg/kg synthetic GnRH buserelin [10].

In the mammalian ovary, AMH inhibits excess recruitment of primordial follicles and decreases the sensitivity of FSH receptors on growing follicles [11,12]. It was found in humans that AMH exerts its regulatory function in part by inhibiting the aromatase that converts androgens to estrogens, thereby counteracting the effect of FSH [13]. In the human ovary, expression of androgen receptor (AR) was detected in follicles from the transitional stage on and with increasing gene levels during follicle development, in parallel with FSH. Androgens are therefore supposed to promote human follicular growth together with FSH, whereas AMH exerts an inhibitory effect [14]; in this species, the FSH-initiated aromatase stimulation leads to increasing estrogen (E2) concentrations, which is supposed to negatively regulate local AMH expression [15]. In cultured human lutein granulosa cells, the expression of AMHRII was down-regulated by LH [16]. No study is available in the international literature concerning the expression and regulation of AMH and AMHRII in feline ovaries.

The serum-AMH concentration is dependent on several factors. In humans, the serum-AMH concentration decreases during menopause, which is a worthful diagnostic tool [17,18]. A decrease with increasing age was also observed in dogs, namely from the 4th year of life on [19]. Similarly in cats, the AMH serum concentration is variable-dependent on age and in addition on the stage of the estrus cycle (13); the highest AMH concentrations were measured at the age of three months, with a significant decrease towards the age of 12 months [20].

During human pregnancy, stable or increasing total AMH serum-concentrations were found to be related to an increased risk for preterm birth [21]. A decrease of serum AMH is normal during human pregnancy [22]; however, reports are variable obviously additionally dependent on women’s age and it is not known whether and how pregnancy changes the bioactivity of AMH [23]. No data are available concerning the serum AMH concentration in pregnant cats.

Our aim was therefore to determine the physiological expression of AMH and its receptor in the feline ovary, and the related serum-AMH concentrations, during normal feline pregnancies and in non-pregnant cats.

## 2. Materials and Methods

### 2.1. Animals and Sample Collection

A total of 33 cats (26 domestic shorthair and 7 domestic longhair cats) with a mean age of 1.03 ± 0.17 years and a weight of 3.36 ± 0.63 were assigned to this study. All cats were brought to the clinic by their owners with the request of spaying for population control, except one cat brought for c-section, and all were healthy at presentation. The gestational age was calculated according to extrafetal and fetal structures assessed by ultrasonographic examination (5 mHz convex transducer; MyLab^TM^Five VET, Esaote, Istanbul, Turkey) (for review: [24]. Parameters before day 30 were inner and exterior chorionic cavity and embryo/fetal length [25]. After day 30, biparietal and body diameter or crown rump length were measured [26]. In non-pregnant controls, the stages of the estrus cycle were determined by cytologic examinations as well as the assessment of steroid hormone concentrations. Vaginal smears were obtained from the anterior vaginal wall with a sterile swab, stained using the Papanicolaou technique and evaluated with a light microscope (Leica Microsystems Inc., Richmond, IL, USA). The cats were divided into groups according to their pregnancy stages and ovarian/placental endocrine activity. Group I (*n* = 3, 23–30 days), embryonal phase; highest progesterone secretion, mostly from the corpora lutea [27]. Group II (*n* = 8, 31–40 days), fetal phase; intraplacental progesterone synthesis increases significantly [28]. Group III (*n* = 4, 41–50 days); intraplacental progesterone concentration decreases, regression of corpora lutea, decreasing plasma progesterone concentration [28]. Group IV (*n* = 6, 51–61 days), preterm/term; placental PGF2α significantly increased, initiating final luteolysis [29]. Group V (*n* = 6, interestrus cats) and group VI (*n* = 6, estrus cats).

All cats underwent ovariohysterectomy under general anesthesia; a blood sample was taken before the operation. Anesthesia was induced with medetomidine (0.01 mg/i.v, Tomidin^®^, Alivira, Polatli, Turkey) and propofol (1%, 6 mg/kg/i.v, Propofol^®^, Fresenius, Istanbul, Turkey) given 10 min after medetomidine administration and maintained with isoflurane (2.0%, Isoflurane^®^, Adeka, Istanbul, Turkey) and oxygen. Immediately after the operations, the uteri and fetuses were macroscopically investigated for pathological lesions and the ovaries were fixed in 10% neutral formalin for 24 h. Blood samples were centrifuged at 1550× *g* for 10 min and the serum samples stored at −80 °C until analyzed.

All uteri obtained after ovariohysterectomy were examined macroscopically and histologically and no abnormalities were determined. Cats in the pregnant groups (I–IV) had an average fetus number of 4.0 ± 1.5.

### 2.2. Serum Progesterone and Oestrogen Measurements

Serum progesterone (P4) and estrogen (E2) concentrations were analyzed using a feline-specific enzyme-linked immunosorbent assay (ELISA; Sunred Biological Technology Co. Ltd., Shanghai, China; cat.nr. 201-28-0243 and 201-28-0206, respectively). The assays were performed according to the manufacturer’s protocol. Analyses were performed concurrently in duplicate. For the P4 assay, 50 μL of standards to the standard wells and 40 μL of serum + 10 μL of P4-antibody to test wells were added. Then, 50 μL of streptavidin-HRP was added to all wells and incubated for 1 h at 37 °C. After washing 5 times, 50 μL of the chromogen solution A, then 50 μL of the chromogen solution B were added to each well and incubated for 10 min at 37 °C away from light. After 50 μL of stopping solution was added to each well, the absorbance of color in the microplate was evaluated in a microplate reader (Infinite F50, Tecan Austria GmbH, Grödig, Austria) and the P4 concentrations of serum were calculated according to standard concentrations. Progesterone ELISA sensitivity: 0.347 ng/mL; assay range: 0.4–300 ng/mL.

For the E2 assay, 50 μL of standards to the standard wells and 40 μL of serum + 10 μL of E2-antibody to test wells were added. Then, 50 μL of streptavidin-HRP was added to all wells and incubated for 1 h at 37 °C. After washing 5 times, 50 μL of the chromogen solution A, then 50 μL of the chromogen solution B were added to each well and incubated for 10 min at 37 °C away from light. After 50 μL of stopping solution was added to each well, the absorbance of color in the microplate was evaluated in a microplate reader (Infinite F50, Tecan Austria GmbH, Grödig, Austria) and the E2 concentrations of serum were calculated according to standard concentrations. Oestrogen ELISA sensitivity: 1.368 pg/mL; assay range: 1.5–400 pg/mL. To validate the ELISA, determination of the intra-assay coefficient of variation (CV), inter-assay CV, recovery, linearity and parallelism was performed. The intra-assay CV was below 10% and inter-assay CV below 12% for both P4 and E2 concentrations.

### 2.3. Serum AMH Measurements

The serum AMH concentrations were measured using an enzyme-linked immunosorbent assay (ELISA) kit (AL-116, Ansh Labs, 445 Medical Center Blvd. Webster, TX, USA) following the manufacturer’s protocol.

Analyses were performed concurrently in duplicate. Briefly, serum or standards and controls (50 μL/well) were incubated with 50 μL of AMH assay buffer in 96-well microtiter plates for 2 h at room temperature (23 ± 2 °C). After washing 5 times, 100 μL of the antibody-biotin conjugate RTU was added to each well and incubated for 1 h at room temperature. After washing 5 times, 100 μL of TMB chromogen solution was added in each well and incubated for 12 min at room temperature. After 100 μL of stopping solution was added in each well, the absorbance of color in the microplate was evaluated in a microplate reader (Infinite F50, Tecan Austria GmbH, Grödig, Austria) and the AMH concentrations of serum were calculated according to standard concentrations. AMH ELISA sensitivity: 0.15 ng/mL; assay range: 0.3–11.9 ng/mL. To validate the ELISA, determination of the intra-assay coefficient of variation (CV), inter-assay CV, recovery, linearity, and parallelism was performed. The intra-assay CV was below 8% and inter-assay CV below 10% for AMH concentrations.

### 2.4. Western Blot Analysis (WB)

To prove the usefulness of the antibodies that are not species-specific, a western blot was performed, using other feline ovaries than for IHC. All ovaries were obtained after routine ovariohysterectomy at the Clinical Unit of Obstetrics, Gynecology and Andrology of the University of Veterinary Medicine Vienna (Austria). The WB analyses were done at the Institute for Histology and Embryology, University of Veterinary Medicine Vienna (A). All tissues were shock frozen in liquid nitrogen followed by immediate resection to avoid protein degradation. To prepare the protein lysates, samples were cut into small pieces and homogenized using a Dounce homogenizer in RIPA lysis buffer (50 mM Tris-HCl pH 7.4, 500 mM NaCl, 0.5% sodium deoxycholate (all Carl Roth GmbH, Karlsruhe, Germany), 1% Nonidet P-40 (Igepal, Sigma Aldrich, Vienna, Austria), 0.1% sodium dodecyl sulfate (Serva), SDS lysis buffer (62.5 mM Tris-HCl, pH 6.8, 50 mM DTT (Carl Roth GmbH, Karlsruhe, Germany), 2% SDS (Merck, Vienna, Austria), 10% glycerol (Serva, Heidelberg, Germany), both supplemented with 1% (*v*/*v*) protease and phosphatase inhibitors (Protease Inhibitor Cocktail and Phosphatase Inhibitor Cocktail 3, both Sigma Aldrich, Vienna, Austria) or TRIzol^®^ Reagent (Thermo Fisher Scientific, Vienna, Austria). Lysates were incubated on ice for 30 min and vortexed occasionally. To shred the DNA, homogenates were pushed through a 20G needle several times and subsequently centrifuged for 15 min at 4 °C using 10.000 rpm. The soluble supernatant fractions were stored at −80°C until further analysis. Protein concentrations were measured using DC™ Protein Assay (Bio-Rad, Vienna, Austria) according to the manufacturer’s recommendations.

Protein extracts (20 µg protein/lane) were separated on 10% polyacrylamide minigels and transferred onto the PVDF membrane (GE Healthcare, Tiefenbach, Austria). To prevent unspecific binding, membranes were blocked in Western Blocking Reagent (Roche, Vienna, Austria; 1:10 in TBST) for 2 h at room temperature followed by overnight incubation with primary antibody at 4 °C. After washing with TBST solution (5 × 8 min at room temperature), the secondary antibody was applied for 30 min at room temperature. As primary antibodies, rabbit polyclonal AMH antibody (GeneTex, Irvine, CA, USA; cat.# GTX129593; dil. 1:500 and 1:1.500) or rabbit polyclonal AMHR2/MISRII antibody (LSBio, Seattle, WA, USA; cat.# LS-B11943, dil. 1:500 and 1:1.500) were used. The Amersham ECL-anti-rabbit IgG peroxidase-linked species-specific whole antibody from donkey (GE Healthcare, Tiefenbach, Austria; cat. # NA934; dil. 1:5000) was used as a secondary antibody. All antibodies were diluted in Western Blocking Reagent (Roche, Vienna, Austria)/TBST (1:10). The signals were visualized using Amersham Western Blotting Detection Reagents (GE Healthcare, Tiefenbach, Austria) and and BioRad ChemiDoc Image System with Image Lab Software (Bio-Rad, Vienna, Austria).

### 2.5. Immunohistochemistry for Ovarian AMH and AMHRII

All ovaries were fixed in 10% neutral formalin for 24 h, then paraffin blocks were prepared. Paraffin-embedded tissue sections, placed on slides coated with 3-aminopropyltriethoxysilane (Sigma, St. Louis, MT, USA), were stained by the streptavidin-biotin-peroxidase complex (SBPC) technique (Histostain Plus kit; Invitrogen, Camarillo, CA, USA) using the following primary antibodies: monoclonal anti-AMH antibody (GTX129593, Genetex, Irvine, CA, USA; reactivity: Human, Mouse) and polyclonal anti-AMHR2 antibody (LS-B11943, LSBio, Seattle, WA, USA; reactivity: Rabbit, Mouse, Dog, Rat, Pig, Horse, Bat, Human). The slides were dried overnight at 37 °C, dewaxed, changing the xylene twice, with a 10-min interval between changes, rehydrated using the graded alcohol series, and placed in distilled water for 10 min. Antigen retrieval was facilitated by heating in citrate buffer (pH 6.0) for 20 min in a microwave oven with a power of 600 W. The slides were dipped in freshly prepared absolute methanol containing hydrogen peroxide (H_2_O_2_) 3% *v*/*v* for 5 min to block endogenous peroxidase activity. After washing with phosphate buffer solution (PBS), all sections were preincubated in 10% normal goat serum for 30 min at room temperature (RT) to block nonspecific binding of the second-step antibody. Sections were incubated with primary antibodies (1:200 dilution for AMH and 1:100 dilution for AMHRII) overnight at +4 °C and were rinsed with PBS. Then the sections were incubated with a broad-spectrum biotin-conjugated second-step antibody (85-9043, Zymed, Sacramento, CA, USA) for 10 min at room temperature (RT) and rinsed in PBS. SBPC was applied for 10 min at RT. Amino ethyl carbazole (AEC, Invitrogen) was used as chromogen in H_2_O_2_ for 10 min (controlled by visual observation with a microscope). The sections were counterstained with Mayer’s hematoxylin for 1 min, rinsed with tap water, and mounted with an aqueous mounting medium (C9368; Sigma-Aldrich, Istanbul, Turkey). Negative controls were prepared by both omitting the primary antibodies and replacing with PBS. Positive and negative control sections of ovary for AMH were used in all immunolabelling procedures. Positive control sections of mouse ovary for AMH and AMHRII were used in all immunolabelling procedures. The distribution of immunoreactivity was examined with a Nikon Eclips E-600 light microscope. The percentage of the total area of AMH and AMHRII immunopositivity were assessed by using a microscopy image analysis system (Bs200P Image Analysis System, BAB software, Ankara, Turkey). A total of 10 fields were chosen and analyzed at ×140 magnification (550.000 μM^2^).

### 2.6. Follicle and Corpus Luteum Counting

After recording the number of corpora lutea macroscopically, the ovaries were bisected longitudinally, embedded in paraffin wax, serially sectioned at 5 μM (50 slices in average per ovary) and stained with hematoxylin and eosin. Every tenth section was selected for observation under a light microscope (Eclipse E600, Nicon, Istanbul, Turkey). Sections were analyzed by light microscopy at ×1000 magnification, and the follicles were counted. To minimize the possibility of counting follicles more than once, only follicles with a visible oocyte nucleolus were recorded. Two independent observers performed the histological evaluations. The follicles were classified into five stages as follows: primordial, primary, secondary, early antral, and antral follicles. An area of 3.500.000 μM^2^ per section was evaluated (5 sections per ovary; 10th, 20th, 30th, 40th, and 50th) at ×60 magnification. Then the average number of follicles and corpora lutea was determined.

### 2.7. Statistical Analyses

Data were analyzed using the statistical package program SPSS Statistics V22.0 (IBM Corporation, Armonk, NY, USA). All data are given as mean ± standard deviation (mean ± SD). Normal distribution and homogeneity of variances were confirmed by Shapiro-Wilks and Levene’s tests, respectively. Comparison of variables between the groups for serum AMH concentrations was carried out by One Way Anowa Tamhane’s T2 test while equal variances were not assumed. The differences of the AMH and AMHRII expression between the groups were determined using One Way Anowa Test (Post Hoc Duncan test). Correlations between ovarian AMH expression and follicle/corpora lutea count were calculated by Pearson’s correlation test. A *p*-value of <0.05 was considered significant.

## 3. Results

### 3.1. Western Blot Analysis

**Figure 1:**
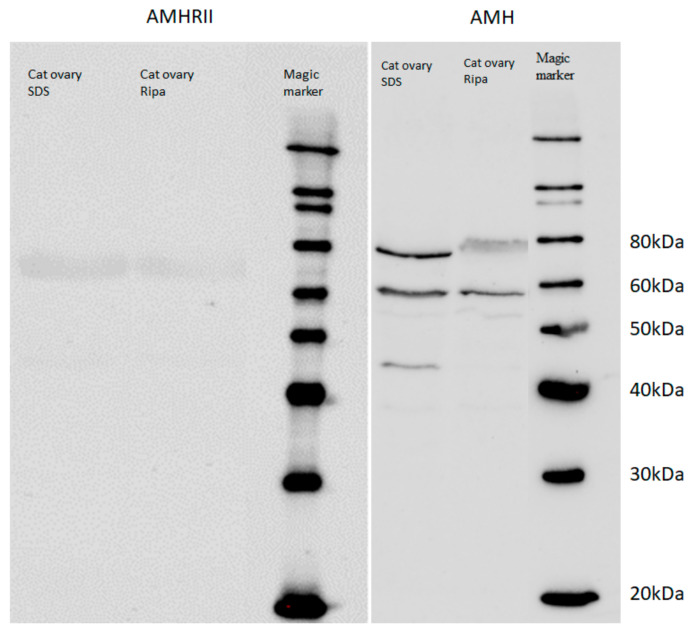
AMH- and AMHRII—Western blot analysis. Western blot detection of the AMH and AMHRII protein—note the bands of about 60–80 kDa in the cat ovary cells. Ripa = Ripa lysis buffer, SDS = SDS lysis buffer. Antibody-dilution: 1:500.

Western blot detection of the AMH protein revealed bands of about 60–80 kDa in the cat ovary samples with the GTX129593 antibody. (Figure 1, Figures S1 and S2) For AMHRII protein, positive bands of different molecular weights (>60 kDa) were seen after incubation with the LS-B11943 antibody.

### 3.2. Follicle and Corpus Luteum Counts

There was no significant difference between groups in terms of follicle counts. However, the number of corpora lutea per group of cats differed significantly (Table 1). The most corpora lutea were counted in group I compared to later stages (group IV) and non-pregnant cats (group V). A few corpora lutea were found in interestrus cats; however, they were considered endocrinologically non-functional since progesterone concentration was basal (Table 1).

### 3.3. Plasma Oestrogen and Progesterone Concentration

**Figure 2:**
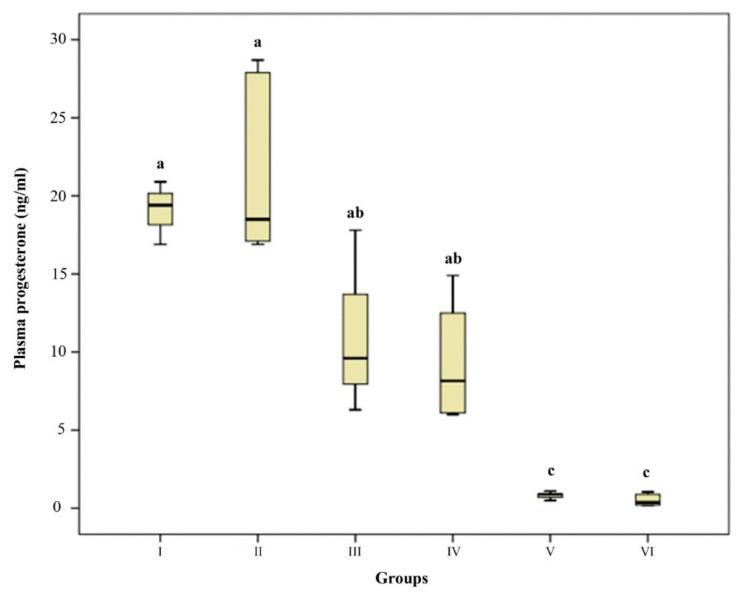
Plasma Progesterone concentration. Box-and-whisker plot. Group I (*n* = 3, 24–29 days), Group II (*n* = 8, 32–40 days), Group III (*n* = 4, 41–46 days), Group IV (*n* = 6, 53–61 days) Group V (*n* = 6, interestrus), Group VI (*n* = 6, estrus). ^a,b,c^ *p* < 0.01.

**Figure 3:**
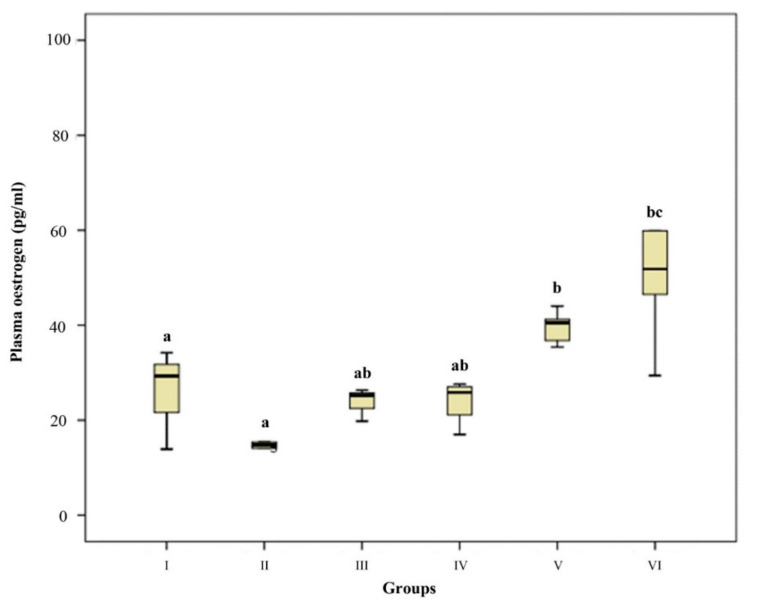
Plasma Estrogen concentration. Box-and-whisker plot. Group I (*n* = 3, 24–29 days), Group II (*n* = 8, 32–40 days), Group III (*n* = 4, 41–46 days), Group IV (*n* = 6, 53–61 days) Group V (*n* = 6, interestrus), Group VI (*n* = 6, estrus). ^a,b^ *p* < 0.05, ^a,c^ *p* < 0.01.

Plasma P4 concentration of GI and GII were significantly higher than GIII (day 41–46) and GIV (day 53–60), and higher than in interestrus and estrous cats (GV and VI) (*p* < 0.01). In GV and GVI, plasma P4 was basal (Figure 2). Plasma E2 concentrations of GV and GVI were significantly higher than in the pregnant groups (GI-II) (*p* < 0.05 and *p* < 0.01). (Figure 3).

### 3.4. AMH Serum Concentration

**Figure 4:**
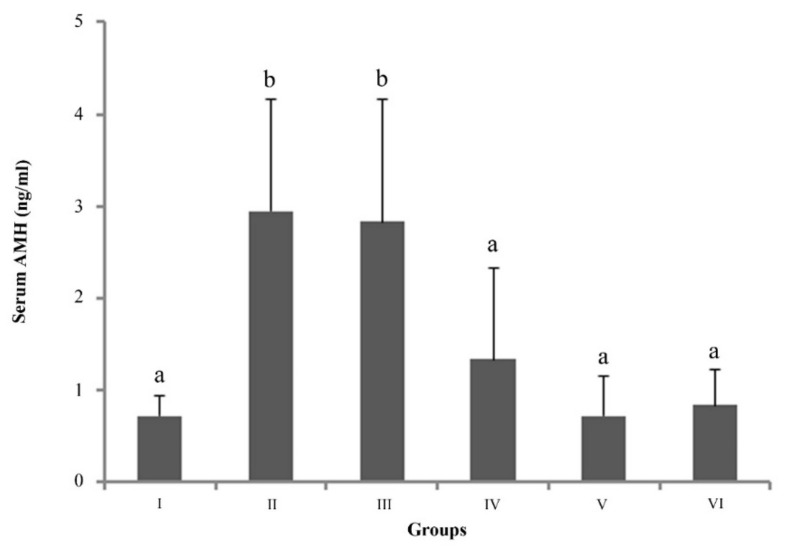
Serum AMH concentration. Group I (*n* = 3, 24–29 days), Group II (*n* = 8, 32–40 days), Group III (*n* = 4, 41–46 days), Group IV (*n* = 6, 53–61 days), Group V (*n* = 6, interestrus), Group VI (*n* = 6, estrus), (mean ± SD).^a,b^ *p* < 0.05.

Serum AMH concentrations for group I, II, III, IV, V and VI were 0.72 ± 0.22 ng/mL, 2.95 ± 1.21 ng/mL, 2.83 ± 1.33 ng/mL, 1.34 ± 0.98 ng/mL, 0.72 ± 0.44 ng/mL and 0.84 ± 0.39 ng/mL, respectively (Figure 4). Serum AMH concentration increased significantly towards mid-gestation, stayed elevated until day 50, and decreased towards term (*p* < 0.05). The serum concentration in group II and III was higher than in all other groups (*p* < 0.05). There was no correlation between AMH serum concentration and fetus number.

### 3.5. Immunohistochemical Findings for AMH

**Figure 5:**
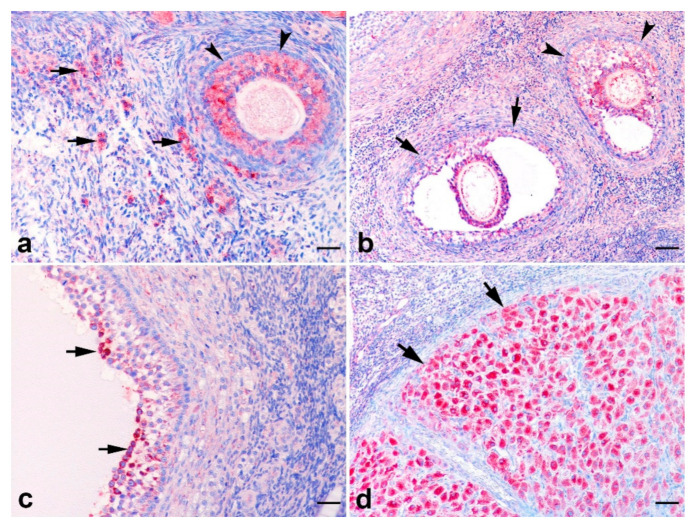
AMH immunopositivity in the feline ovary. (**a**) AMH immunopositivity in granulosa cells of secondary follicles (arrow heads) and interstitial luteal cells (arrows); (**b**) AMH immunopositivity in granulosa cells of early antral follicles (arrow heads) and antral follicles (arrows), (**c**) AMH immunopositivity in mural granulosa cells of large antral follicles (arrows); (**d**) AMH immunopositivity in luteal cells of corpus luteum (arrows). SABP immunostaining, AEC chromogen, Mayer’s hematoxylin counterstaining, (**a**,**c**) ×280 and (**b**,**d**) ×140. Scale bar: (**a**,**c**) = 30 μM and (**b**,**d**) = 60 μM.

In the ovaries, AMH immunopositivity was observed in the cytoplasm of granulosa cells. There was no staining in the flattened granulosa cells of primordial follicles though oocyte cytoplasm expressed AMH, but this staining was evaluated as nonspecific background staining. No or very weak AMH expression was observed in cuboidal granulosa cells of primary follicles. In secondary (Figure 5a), early antral (Figure 5b), and small antral follicles, AMH immunopositivity was very strong. Most of the granulosa cells of these follicles expressed AMH. Moderate or strong staining was observed in nearly all granulosa cells of small antral follicles in which the diameter was less than 600 μM. The immunopositivity in mural granulosa cells found in limited number in large antral follicles was variable (Figure 5c). The cumulus granulosa cells of large antral and small antral follicles showed intense AMH expression. Immunopositivity in large antral follicles was lower compared to particularly secondary, early antral, and small antral follicles. Additionally, in all corpora lutea of all cats, moderate or strong staining was observed (Figure 5d). Similarly, strong immunopositivity was detected in interstitial cells.

**Figure 6:**
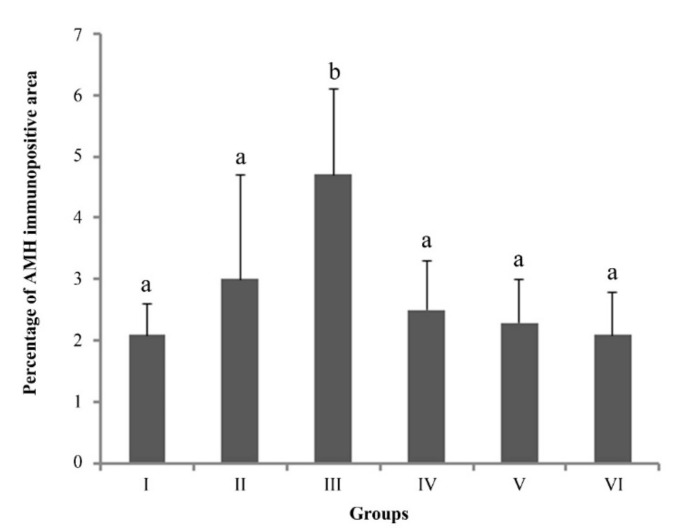
Ovarian AMH expression. Group I (*n* = 3, 24–29 days), Group II (*n* = 8, 32–40 days), Group III (*n* = 4, 41–46 days), Group IV (*n* = 6, 53–61 days), Group V (*n* = 6, interestrus), Group VI (*n* = 6, estrus). ^a,b^ *p* < 0.05.

When the percentage of AMH immunopositive areas was compared between groups, the percentage was higher in group III (day 41–50) than in all other stages, and in comparison to interestrus and estrus cats (*p* ˂ 0.05) (Figure 6).

In non-pregnant controls (V), positive correlations between ovarian AMH, and antral follicles (*r* = 0.931, *p* ˂ 0.05) and total antral follicles (*r* = 0.858, *p* ˂ 0.05) were assessed. This was not observed in pregnant cats (I–IV); however, during mid-gestation (Group II), a positive correlation between the number of corpora lutea and the ovarian AMH expression (*r*^2^ = 0.832, *p* ˂ 0.05) was determined.

### 3.6. Immunohistochemical Findings for AMHRII

**Figure 7:**
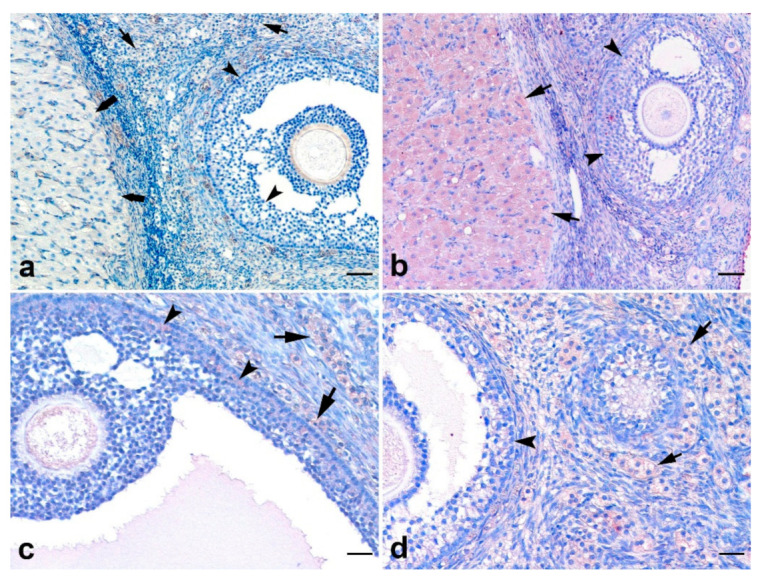
AMHRII immunopositivity in the feline ovary. (**a**) negative control, AMHRII negative luteal cells in corpus luteum (thick arrows), interstitial luteal cells (arrows) and granulosa cells of follicle (arrow heads); (**b**) AMHRII immunopositivity in granulosa cells of early antral follicles (arrow heads) and corpus luteum (thick arrows); (**c**) AMHRII immunopositivity in mural granulosa cells of large antral follicles (arrow heads) and interstitial luteal cells (arrows); (**d**) AMHRII immunopositivity in granulosa cells (arrow head) and interstitial luteal cells (arrows). SABP immunostaining, AEC chromogen, Mayer’s hematoxylin counterstaining, (**a**–**c**) ×140, (**d**) ×280. Scale bar: (**a**,**b**) = 60 μM and (**c**,**d**) = 30 μM.

**Figure 8:**
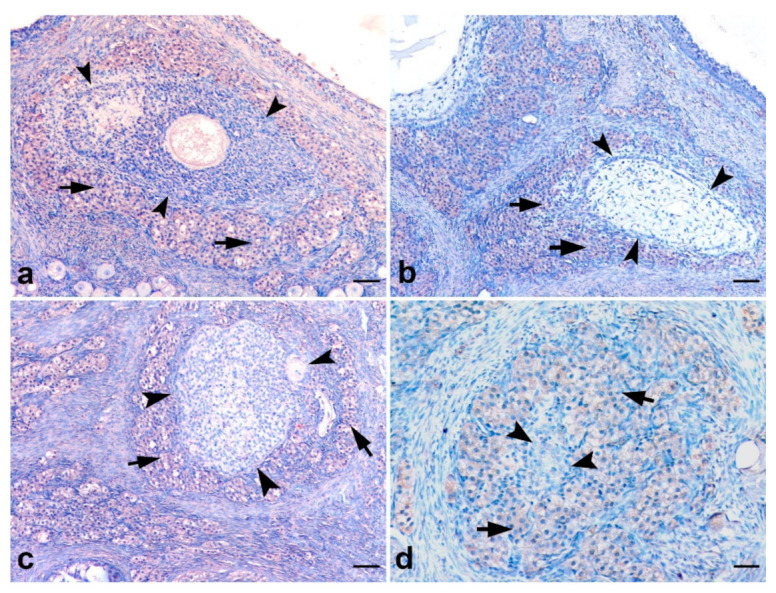
AMHRII immunopositivity in the feline ovary. (**a**–**d**); atretic follicles in different stages (arrow heads) and AMHRII immunopositive luteal cells surrounding these follicles (arrows) SABP immunostaining, AEC chromogen, Mayer’s hematoxylin counterstaining, (**a**–**c**) ×140, (**d**) ×280. Scale bar (**a**–**c**) = 60 μM and (**d**) = 30 μM.

Negative controls did not show immunopositivity (Figure 7a). The expression of AMHRII was diffuse homogenous but weak in all luteal and interstitial cells in the ovary as well as granulosa cells in few preantral and antral follicles (Figure 7b–d). AMHRII immunopositivity was additionally detected in corpus luteum cells. On the other hand, the expression of the receptor was strong in the interstitial cells surrounding the follicles that underwent atresia (Figure 8a–d).

No difference was observed between pregnancy stages for AMHRII expression. However, AMHRII expression was higher in all pregnant groups compared to estrus cats (*p* ˂ 0.05), and in group I (day 24–29) and III (day 41–46), the AMHRII proportion of immunopositive areas was higher than in interestrus cats (*p* ˂ 0.05).

## 4. Discussion

To the best of our knowledge, this is the first report on serum AMH concentrations and the expression of AMH and its type 2 receptor in the ovary of pregnant domestic cats.

When talking about circulating AMH, two forms must be differentiated, the pro-hormone proAMH not binding to AMH receptors, and the receptor-affin AMH. Nevertheless, in most studies, the course of the total serum-AMH concentrations is described without this differentiation. The results of our study demonstrate that the total-serum AMH concentration increased after day 30, was elevated until day 50, then decreased towards term; the course of the serum-AMH concentration paralleled the degree of AMH expression in the ovaries. The levels of progesterone were consistent with the physiological endocrinology of feline pregnancy.

Studies on serum AMH levels during human pregnancy revealed a drop from the first to the second trimester [30,31,32] and from the second to the third trimester [30,32,33]; thus, in pregnant women, blood AMH concentrations decline with gestational age. The decrease in AMH concentrations during human pregnancy is thought to be related to ovarian suppression. The AMH serum concentration is related to the continuous FSH-independent non-cyclic growth of small follicles, and during human pregnancy, the decrease in serum AMH surely reflects a decrease in follicular maturation. In cats, follicular maturation is known to occur during pregnancy, which might explain the increase in AMH serum concentration between day 30 and 50 of gestation.

When the course of serum-AMH concentration during human pregnancy was related to age, only women ≥35 years showed no significant differences in AMH concentrations between all trimesters. However, there was a significant drop from the first to the second trimester as well as from the second to the third trimester in the age groups of ≤27 years and 28–34 years [32]. Gerli and co-workers [25] stated a significant decrease in the concentrations during the third trimester of human pregnancy independent of maternal age. As AMH concentration is known to be age-dependent not only in humans but also in the cat [20], this study only included cats with a mean age of 1.03 ± 0.17 years.

The results of [34] determined that plasma AMH concentration is correlated to the occurrence of ovulation in ewe lambs after administration of an ovarian stimulation treatment and this finding was thought to be associated with the reflection of the population of gonadotropin-responsive follicles or in other words ovarian activity. Additionally, high AMH concentrations were detected in women with polycystic ovarian syndrome [35] and this is thought to be due to excess numbers of pre-antral and small antral follicles that may not have reached the maturity that will respond to FSH, therefore resulting in an increase in AMH concentrations [36]. Elevated levels of AMH are known to suppress FSH secretion, but not LH secretion in anterior pituitaries of post-pubertal heifers [37]. It is well known that AMH regulates follicle growth and eliminates FSH sensitivity. In our study, the significantly higher serum AMH concentrations in GII (31–40 days) and III (41–50 days) compared to the other groups may be attributed to the above-mentioned effect of AMH on FSH levels, namely that no FSH activity is needed during these stages of cat pregnancy. Previous investigation on the physiological dynamics of the cat pregnancy revealed that plasma progesterone concentrations reached a peak on days 30 to 35 of pregnancy, decreased slowly afterwards, and was below 1 ng/mL during parturition [38]. In the present study, high AMH concentrations between day 30 and 50 are supposed to exert an inhibitory effect on follicular growth, thus it presumably suppresses ovarian follicular responses to gonadotropins during that stage of pregnancy in the cat. Additionally, it has been demonstrated that GnRH neurons in mice and humans express AMH receptor, and in these species, AMH increases GnRH dependent LH secretion [39]. In the cat, LH is needed for the maintenance of pregnancy particularly in the second half of pregnancy. Thus, our results implicate an intriguing hypothesis that increased circulating levels of AMH in pregnant cats might stimulate LH secretion; however, since LH was not measured, this must be proven.

In this study, the protein expression of AMH and AMHRII was co-localized in the ovaries of pregnant and non-pregnant cats, which has not been investigated before. Since in pregnant and non-pregnant cats, vesicular follicles can be observed during all stages of the luteal phase, similarly in the interestrus phase [40], this was expected. The ovarian AMH expression correlated significantly with antral follicle count, as was shown in other species. Also comparable to other species like rats, birds, and humans [41,42,43], AMH immunoreactivity was mainly present in the cumulus granulosa cells of small and large antral follicles, and in corpus luteum cells. In our cats, the expression was without any difference between pregnancy stages, pregnant and non-pregnant cats, or interestrus and estrus cats concerning the sites of expression. Similarly, no change during the estrus cycle has also been described in rats [41]. In our study, the only detectable difference concerned the degree of expression, with more intense immunostaining in pregnant cats between day 41 and 50 than in other groups. In hens, higher expression of AMH and AMHRII was detected in ovaries during reproductive quiescence than in ovaries from layer hens [42]. Authors hypothesized that this might point towards a protecting effect of AMH for the ovarian reserve. Whether this applies similarly in cats remains to be investigated.

The AMHRII was mostly in close co-localization with AMH, indicating an autocrine action of AMH via its receptor, as described in rats [41]. We in addition detected high expression in interstitial cells surrounding follicles that underwent atresia. In rats, decreasing gene expression of both AMH and AMHRII was assessed in follicles undergoing atresia, probably indicating a gene loss; however, AMHRII was longer detectable in atretic follicles, probably as a result of suppression of follicular maturation. In the human ovary, similar sites of expression of AMHRII were found as in our cat ovaries, with the exception that in cats, AMHRII was additionally expressed in corpus luteum cells and granulosa cells of atretic follicles [43].

## 5. Conclusions

We conclude that the AMH and AMHRII expression in the feline ovary is comparable to other species, whereas the course of serum AMH concentration is species-specific and comparable to the rat. The higher serum AMH concentration in pregnant cats in comparison to non-pregnant cats is probably related to ovarian activity during feline mid-pregnancy. Whether AMH exerts an additional extragonadal effect on the hypothalamic-pituitary-gonadal axis, deserves further investigation.

## Figures and Tables

**Table 1:** Number of ovarian follicles and corpora lutea per group.

Follicles	Group I	Group II	Group III	Group IV	Group V	Group VI
Primordial	268.6 ± 104.3	335.7 ± 168.1	185.2 ± 84.7	398.8 ± 165.6	350.5 ± 217.7	246.6 ± 33.1
Primary	11.7 ± 7.8	8.9 ± 3.4	5.2 ± 3.9	8.6 ± 3.0	10.1 ± 6.5	8.3 ± 6.1
Secondary	7.0 ± 3.4	7.0 ± 5.9	4.4 ± 3.1	6.0 ± 4.3	8.6 ± 3.2	4.8 ± 2.7
Early antral	2.2 ± 1.8	2.2 ± 1.1	1.7 ± 1.4	2.8 ± 2.1	2.7 ± 1.7	1.8 ± 0.9
Antral	6.6 ± 4.1	9.2 ± 2.9	5.5 ± 3.0	9.7 ± 5.0	8.4 ± 2.2	5.3 ± 2.1
Total follicles	316.2 ± 135.0	363.1 ± 169.6	177.2 ± 63.5	426.0 ± 167.9	380.5 ± 218.4	267.0 ± 33.6
Corpus luteum	4.25 ± 2.25 ^a^	2.79 ± 0.94 ^ab^	3.50 ± 0.89 ^a^	1.50 ± 0.85 ^bc^	0.38 ± 0.92 ^c^	ND

Group I (*n* = 3, 24–29 days), Group II (*n* = 8, 32–40 days), Group III (*n* = 4, 41–46 days), Group IV (*n* = 6, 53–61 days) Group V (*n* = 6, interestrus), Group VI (estrus *n* = 6). ND = not detected. ^a,b,c^ *p* < 0.05.

## Data Availability

Not applicable.

## Supplementary Materials

The following are available online at https://www.mdpi.com/article/10.3390/ani12070877/s1, Figure S1: AMH Western Blot, Figure S2: AMHRII Western Blot.
