# Supplementary figures and images for: Expression of Anti-Müllerian Hormone and Its Type 2 Receptor in the Ovary of Pregnant and Cyclic Domestic Cats
Source: Animals (Basel). 2022 Mar 30;12(7):877. doi: 10.3390/ani12070877 (PMC8997022; doi:10.3390/ani12070877)

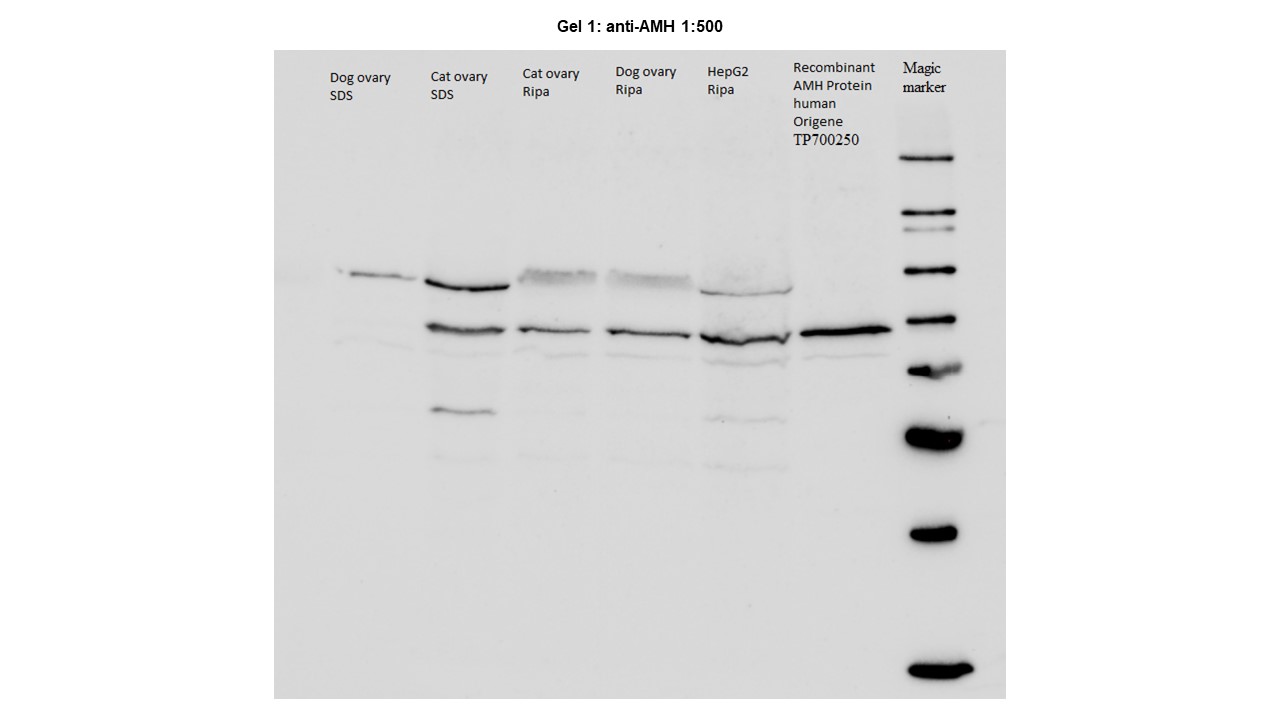

Supplement: Supplementary file 1 [file animals-12-00877-s001.zip › Supplementary/Figure S1. AMH western blot.jpg]

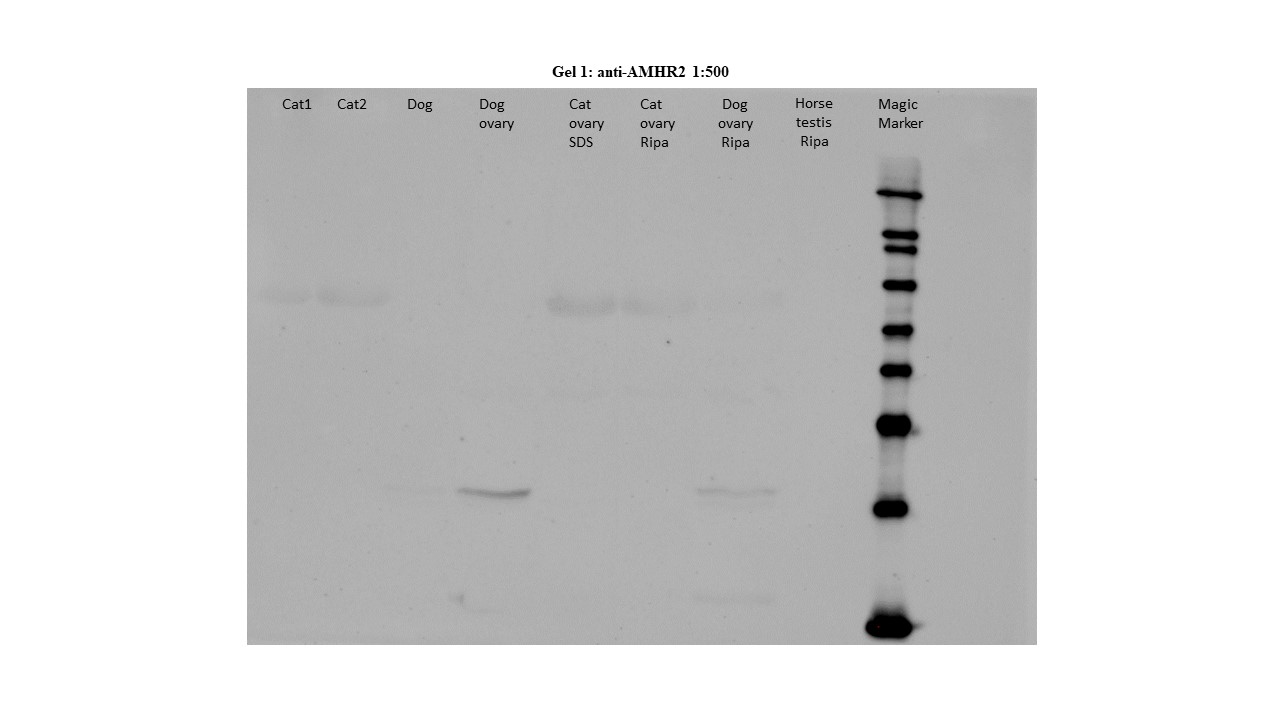

Supplement: Supplementary file 1 [file animals-12-00877-s001.zip › Supplementary/Figure S2. AMHRII western blot.jpg]
